# Identification of genomic aberrations in hemangioblastoma by droplet digital PCR and SNP microarray highlights novel candidate genes and pathways for pathogenesis

**DOI:** 10.1186/s12864-016-2370-6

**Published:** 2016-01-14

**Authors:** Ruty Mehrian-Shai, Michal Yalon, Itai Moshe, Iris Barshack, Dvorah Nass, Jasmine Jacob, Chen Dor, Juergen K. V. Reichardt, Shlomi Constantini, Amos Toren

**Affiliations:** Pediatric Hemato-Oncology, Edmond and Lilly Safra Children’s Hospital and Cancer Research Center, Sheba Medical Center, Tel Hashomer affiliated to the Sackler School of Medicine, Tel-Aviv University, Tel Aviv, Israel; Sackler Faculty of Medicine, Tel Aviv University, Tel Aviv, Israel; Institute of Pathology, Sheba Medical Center, Tel Hashomer, Israel; Division of Tropical Health and Medicine, James Cook University, Townsville, QLD Australia; Department of Pediatric Neurosurgery, Dana Children’s Hospital, Tel-Aviv-Sourasky Medical Center, Tel-Aviv, Israel

**Keywords:** Hemangioblastoma, CGH, digital PCR, cancer

## Abstract

**Background:**

The genetic mechanisms underlying hemangioblastoma development are still largely unknown. We used high-resolution single nucleotide polymorphism microarrays and droplet digital PCR analysis to detect copy number variations (CNVs) in total of 45 hemangioblastoma tumors.

**Results:**

We identified 94 CNVs with a median of 18 CNVs per sample. The most frequently gained regions were on chromosomes 1 (p36.32) and 7 (p11.2). These regions contain the *EGFR* and *PRDM16* genes. Recurrent losses were located at chromosome 12 (q24.13), which includes the gene *PTPN11*.

**Conclusions:**

Our findings provide the first high-resolution genome-wide view of chromosomal changes in hemangioblastoma and identify 23 candidate genes: *EGFR, PRDM16, PTPN11, HOXD11, HOXD13, FLT3, PTCH, FGFR1, FOXP1, GPC3, HOXC13, HOXC11, MKL1, CHEK2, IRF4, GPHN, IKZF1, RB1, HOXA9,* and micro RNA, such as hsa-mir-196a-2 for hemangioblastoma pathogenesis. Furthermore, our data implicate that cell proliferation and angiogenesis promoting pathways may be involved in the molecular pathogenesis of hemangioblastoma.

## Background

Hemangioblastomas (HB) are highly vascular tumors, which account for approximately 3 % of all tumors of the central nervous system (CNS) [1]. It occurs in a subset of CNS locations, including the cerebellum (37 %), brainstem (10 %), and spinal cord (50 %) [1]. They are classed as grade one tumors under the World Health Organization's classification system. While most of these tumors are low grade and benign, some hemangioblastomas can present aggressive and occasionally malignant behavior. Hemangioblastomas occur as sporadic tumors (75 %) or as a manifestation of an autosomal dominantly inherited disorder, von Hippel-Lindau (VHL) disease (25 %) [2].

VHL hemangioblastomas are most commonly caused by germline exon deletions or truncating mutations [3] of the Von Hippel-Lindau *(VHL*) tumor-suppressor gene. The VHL protein, which is the critical part of a ubiquitin ligase protein complex that binds to the hypoxia-inducing factors HIF-1 and HIF-2 transcription factors and targets them for ubiquitination and proteosomal degradation. Dysregulation of this VHL-associated function causes increased expression of a variety of growth factors, including erythropoietin, PDGF, VEGF and TGF. Upregulation of these factors may lead to angiogenesis and tumorigenesis. Additional mechanisms of tumorigenesis have been described outside of the HIF pathway, including alterations in microtubule binding and stabilization, abnormal extracellular matrix composition as well as apoptosis and transcription regulation [4]. For most VHL disease related hemangioblastomas, the inactivation or loss of both alleles of the VHL gene is required. In addition to the phenotypic variability associated with allelic heterogeneity, genetic modifiers may influence the phenotypic expression of VHL disease. Allelic variants in the *CCND1, MMP1* and *MMP3* genes have been reported to influence hemangioblastoma development [5]. This reiterates the need for elucidating other genetic alterations specific for hemangioblastoma beside the hits of *VHL* gene. Moreover, in a subset of tumors including mostly sporadic hemangioblastomas, the genetic pathways involved in tumorigenesis have not been defined yet [6].

Copy number variants (CNVs) are alterations of DNA sections in result of genomic deletions (fewer than the normal number) or duplications (more than the normal number) on certain chromosomes and are common to many human cancers. Comparative genomic hybridization (CGH) by single nucleotide polymorphism (SNP) arrays is a cutting edge technology that allows characterization of CNVs. SNP array karyotyping provides genome-wide assessment of copy number and loss of heterozygosity (LOH) in one assay. SNP array platforms, such as Affymetrix SNP 6.0 (Affymetrix, Santa Clara, CA, USA), often identify amplifications/deletions at a single gene level, which could not have been accomplished by previous methods. Thus, modern SNP arrays offer a powerful method for the discovery of oncogene and tumor suppressor gene involvement in tumors, as well as for improved cancer classification [7].

In contrast to surveillance of genome wide alterations by CGH arrays it is possible to directly quantify the absolute copy number of specific DNA loci by Droplet Digital PCR (ddPCR). In ddPCR, target sequences are amplified by PCR and the reaction products are partitioned into droplets and amplified to endpoint with TaqMan probes as in qPCR, then their concentrations are determined based on the number of fluorescently positive and negative droplets in a sample well. The absolute number of target and reference DNA molecules is calculated and provides the target copy number variation (CNV) [8].

In the present study we used high-resolution SNP arrays for the first time to genome wide analysis of aberrations in hemangioblastomas aiming at the identification of novel pathogenetic mechanisms and possible targets for rational therapy. We validated the main reoccurring genetic changes by ddPCR highly precise quantification.

## Methods

### Study population

A total of 44 hemangioblastoma samples were used for the present study. Thirteen frozen samples obtained from The Sourasky Medical Center, Tel Aviv, Israel were used for the CGH analysis. Additional 32 formalin fixed paraffin embedded (FFPE) samples from Sheba Medical Center, Tel Hashomer, Israel were used as validation group. The study was approved by the ethical review boards of both Sheba and Tel Aviv Sourasky Medical Centers and was consistent with the declaration of Helsinki including informed consents. Clinical parameters, such as sex, age at diagnosis, and pathologic classification were collected from patient records. Clinical information of the patient’s cohort is outlined in Table 1.

**Table 1:** Cohort characteristics

Characteristic	Frozen	Paraffin
Average age	48.5	53.5
Median age	51	53
Spinal samples	4	3
Brain samples	8	29
Total number	12	32

### CGH analysis

DNA was purified from frozen tissues using DNeasy (Qiagen Inc., Valencia, CA). One sample of pooled normal genomic DNA, provided by Affymetrix, was used as experimental positive control. 250 ng of genomic DNA was digested with *Nsp*I (New England Biolabs, Inc) and then ligated to Nsp adaptors. The adaptor-ligated DNA fragments were amplified, fragmented using DNase I, end labelled with a biotinylated nucleotide, and hybridized to a human cytoscan HD array (Affymetrix) at 50 °C for 17 h. After hybridization, the arrays were washed, stained, and finally scanned with a GeneChip scanner 3000 (Affymetrix). All procedures were performed according to the manufacturer’s protocols. Array experiments were performed using the high-resolution Affymetrix CytoScan HD microarray (Affymetrix, Inc, Santa Clara, CA) containing 2,696,550 markers of which 1,953,246 are non-polymorphic markers and 750,000 SNPs with over 99 % accuracy to detect accurate breakpoint estimation as well as loss of heterozygosity (LOH) determination. This chip covers 340 International Standards for Cytogenomic Arrays (ISCA) constitutional genes, 526 cancer genes (99.6 %) and 36,121 RefSeq genes. The chip uses marker intervals of 25 markers / 100 kb. Analysis of CEL files from the Affymetrix CytoScan HD Array or Cytogenetics Whole-Genome 2.7 M Array was done with the Chromosome Analysis Suite (ChAS) software for cytogenetic analysis. Signal processing was done by Signal Covariate Adjustment, Fragment Correction, Dual Quantile Normalization and PLIER signal summarization. Dual Quantile Normalization was done to equalize each array’s intensity distribution copy number and SNP probes separately. For SNP markers, multiple probes for each allele were summarized to single values. Copy number (CN) was calculated by hidden Markov model copy number segments after log2 calculation, high pass filter image correction, log2 ratio covariate adjustment and systematic residual variables removal. The baseline for CN = 2 (normal autosomal copy number state) was established and used by the analysis software by Affymetrix company using a set of 380 phenotypically normal individuals named as reference. The reference sample includes 186 females and 194 male. For chromosome X, only females were used and for chromosome Y only males were used. Log2 ratios for each marker are calculated relative to the reference signal profile. Results of the summarized Data (CYCHP files) were viewed as chromosomal aberrations in table and graphical formats. We also added visual inspection of probe performance for altered segments. Reference intensity intended to represent the copy normal state (typically 2). Log ratios above 0 mean CN gain, log ratios below 0 mean CN loss and log ratios around 0 represent no change. Abnormal DNA copy numbers are identified automatically using 25 markers for loss/50 markers for gains.

### VHL sequencing

To screen the *VHL* gene for mutations in our cohort, we performed direct sequencing of the coding region. Exons 1, 2 and 3 of the *VHL* gene and their immediately flanking sequences were amplified by PCR as described previously [9]. The PCR amplification products were purified by using the QIAquick PCR Purification Kit (Qiagen), according to the manufacturer’s instructions. The amplification primers were used as primers in the sequencing reactions, except for exon 1, for which we designed a new cycle sequencing primer (5′CGAAGATACGGAGGTCGA3′). Cycle sequencing was performed using the ABI PRISM Big Dye Terminator Cycle Sequencing Ready reaction kit (Applied Biosystems, Foster City, CA, USA), followed by isopropanol precipitation. The fragments were sequenced by automated sequencing analysis on an ABI Prism 377 sequencer (Applied Biosystems).

### Droplet digital PCR

Copy Number validation was done on all samples, frozen and paraffin embedded, hemangioblastoma biopsies. Genomic DNA was purified using QIAamp DNA mini (Qiagen). Copy number variation (CNV) test was performed by droplet digital PCR (ddPCR) as previously described [10]. In short, 16 ng of genomic DNA samples were added to 2xddPCR supermix (Bio-Rad) with final concentration of 500nM of each primer and 250nM probe in duplex of the tested gene and RNaseP. RNaseP served as a CNV = 2 reference gene. Probes for the tested genes contained a FAM reporter and RNaseP contained HEX. The genomic DNA and PCR reaction mixtures were partitioned into an emulsion of approximately 20,000 droplets using the QX100 droplet generator (Bio-Rad, USA). The droplets were transferred to a 96-well PCR plate, heat sealed, and placed in a conventional thermal cycler. Thermal cycling conditions were: 95oC for 10 min followed by 40 cycles of 94oC for 30 s, 60oC for 60 s and one cycle of 98oC for 10 min and finally 4oC hold. Following PCR, the plates were loaded into QX100 droplet reader (Bio-Rad) and the CNV value was calculated using Quantasoft software (Bio-Rad, USA). Primers and probes for selected areas enlisted in Table 2 were designed using Primer Express software (PE Corp, USA) and specificity was verified using NCBI BLAST online tool (National Library of Medicine, USA). RNaseP primers and probes were obtained from Bio-Rad.

**Table 2:** Digital PCR primers and probes for selected areas

Gene	Oligonucleotides	Sequence (5′-3′)	reporter
*PTPN11*	Forward primer	TTAGAGACAGGGTCCCACTCTTG	-
	Reverse primer	GCTTGAGGATGCAGTAAGCTATGA	-
	Probe	CCTGGCTGGAGTGCAGTGGCGT	FAM
*CHECK2*	Forward primer	CATTTTTCTCTTAGTATCTTTCTGGGAAT	-
	Reverse primer	CATTTCTGAGCCCAGCAATACA	-
	Probe	TCACAATCCAGGGCTACAGTAAGACCCATG	FAM
*PTCH1*	Forward primer	GCGTGCGAAGGTGGAGACT	-
	Reverse primer	TCATTGGCCTCCCACTTGA	-
	Probe	TGTCTTCTCCCCCATGTCGG	FAM
*EGFR*	Forward primer	AGGAGGAACAACGTGGAGACA	-
	Reverse primer	GAGACACCGGAGCCACAGA	-
	Probe	CCCAGAGGTGGAACGTTGGCCC	FAM
*RNaseP*	Forward primer	GATTTGGACCTGCGAGCG	-
	Reverse primer	GCGGCTGTCTCCACAAGT	-
	Probe	CTGACCTGAAGGCTCT	Hex

## Results

### SNP array profiling identifies recurrent CNVs

**Fig. 1:**
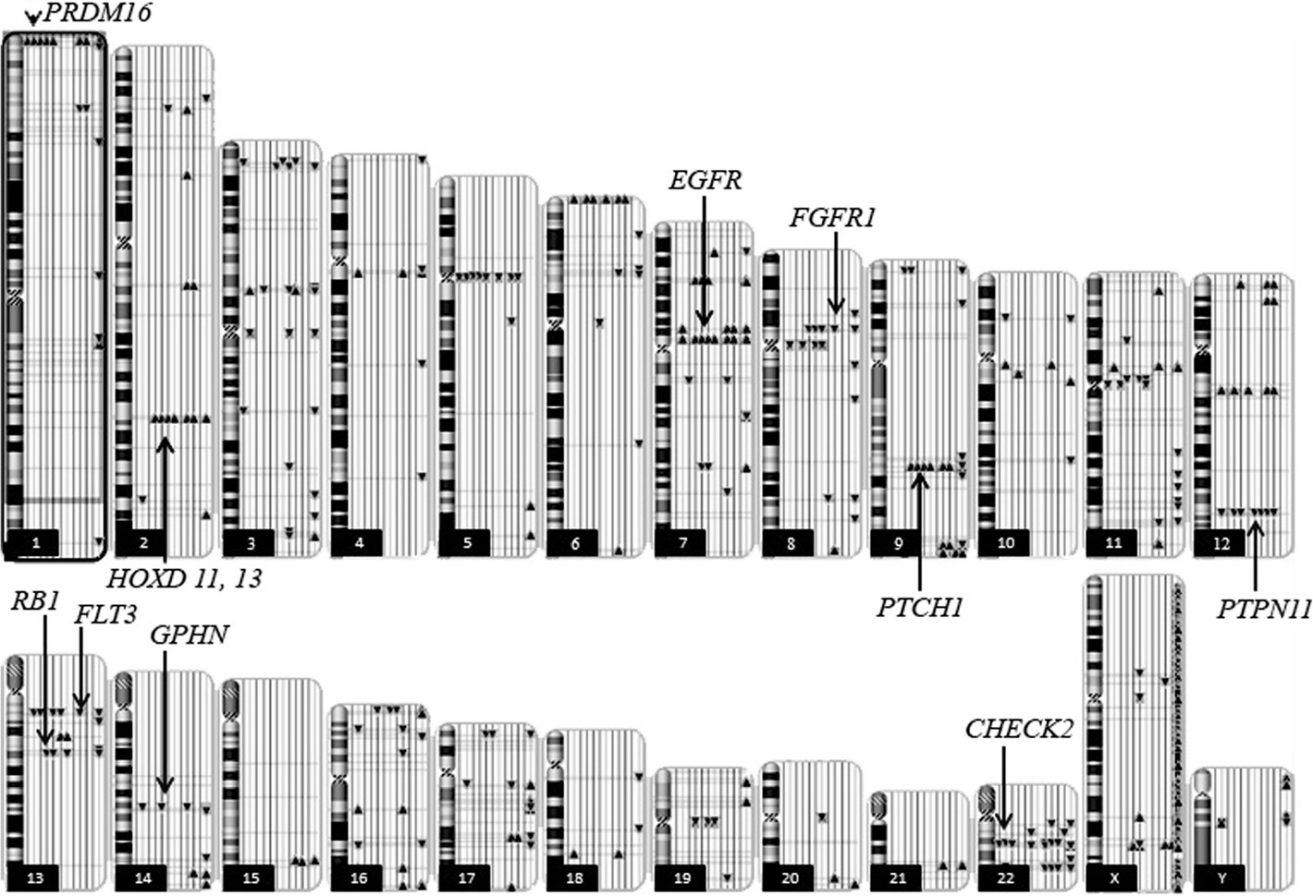
Ideogram illustrating genome-wide distribution of DNA amplifications and deletions (copy number variation) on each chromosome. Each line next to the chromosome represents one sample. Gains in copy number are represented by triangles pointing upwards and losses in copy number are represented by triangles pointing downwards with indication of gene in that region. Chromosome numbers are indicated in boxes

**Fig. 2:**
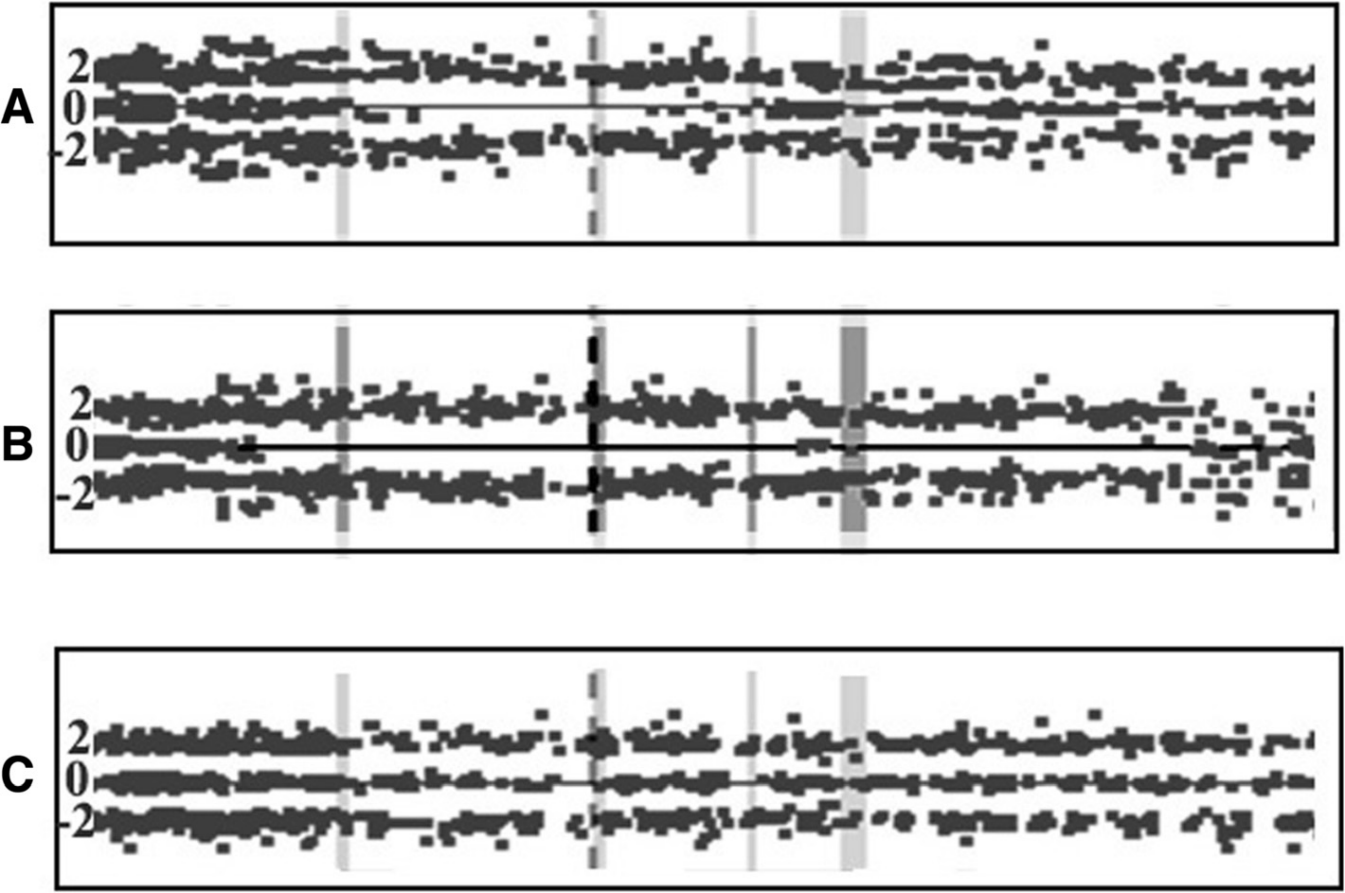
*CHECK2* copy number state. *CHECK2* locus is labeled by centrally located data track with dashed line. The data point scores form a trinomial distribution about the values 2, 0 and −2, where values around 0 represent heterozygous SNPs, while homozygous SNPs have a value of approximately 2 or −2. LOH is located on genomic region with a scarcity of heterozygous SNP calls. **a** Sample 6 and (**b**) Sample 13 exhibit LOH of *CHECK2* region, (**c**) The control sample exhibits normal copy number state

The systematic genome-wide gain and loss segments based CNVs data detected by SNP profiling in hemagioblastoma is detailed in the karyoview (Fig. 1). We identified 94 CNVs with a median of 18 CNVs per sample. Twenty-three of them involved noncoding regions in centromeres that are known to harbor spurious CNVs, most of them were less than 100 Kb long, and 56 were found in at least two samples. The most frequently gained regions were on chromosomes 1 (p36.32) and 7 (p11.2). These regions contain the *PRDM16* and *EGFR* genes, respectively. Recurrent losses were located at chromosome 12 (q24.13) which includes the *PTPN11* gene. Pathway analysis revealed that *EGFR*, Notch and HedgeHog signaling were the most frequently altered pathways promoting angiogenesis and proliferation. *Mir 551a* (part of *PRDM16*) gain was detected in seven samples, *miR-196a-2* gain was observed in five samples and *miR-196b* gain was detected in four samples. The list of recurrent CNVs found in at least five specimens (Fisher exact test *p*-value < 0.05), including type of alteration, involved chromosome, cytobands, and overlapping genes/miRNAs according to the RefSeq database is provided in Table 3. Two samples had LOH affecting the *CHECK2* region (Fig. 2).

**Table 3:** Frequent chromosome losses and gains involving single genes

Number of samples	Type	Chromo some	Band	Gene	miRNA	GV
7	Gain	7	p11.2	*EGFR*		-
7	Gain	1	p36.32	*PRDM16*	hsa-mir-551ahsa-mir-551a	v
7	Loss	12	q24.13	*PTPN11*		-
7	Gain	2	q31.1	*HOXD11, HOXD13 HOHOHOXD13 HOXD11HOXD13*		v
6	Loss	13	q12.2	*FLT3*		-
6	Gain	9	q22.32	*PTCH*		v
5	Loss	8	p11.22	*FGFR1*		-
5	Loss	3	p13	*FOXP1*		-
5	Gain	X	q26.2	*GPC3*		-
5	Gain	12	q13.13	*HOXC13, HOXC11*	hsa-mir-196a-2	v
5	Loss	22	q13.1	*MKL1*		v
5	Loss	22	q12.1	*CHEK2*		v

The column “Number of samples” represents the number of patients with a particular genomic abnormality. For each variation the type of variation (gain or loss), location on chromosome and band, the gene located in this locus and miRNAs located in this locus are enlisted. Variants reported in the Database of Genomic Variants (DGV) database in genes are denoted as “v” and if none reported as “-” under the column DGV

### VHL status

**Fig. 3:**
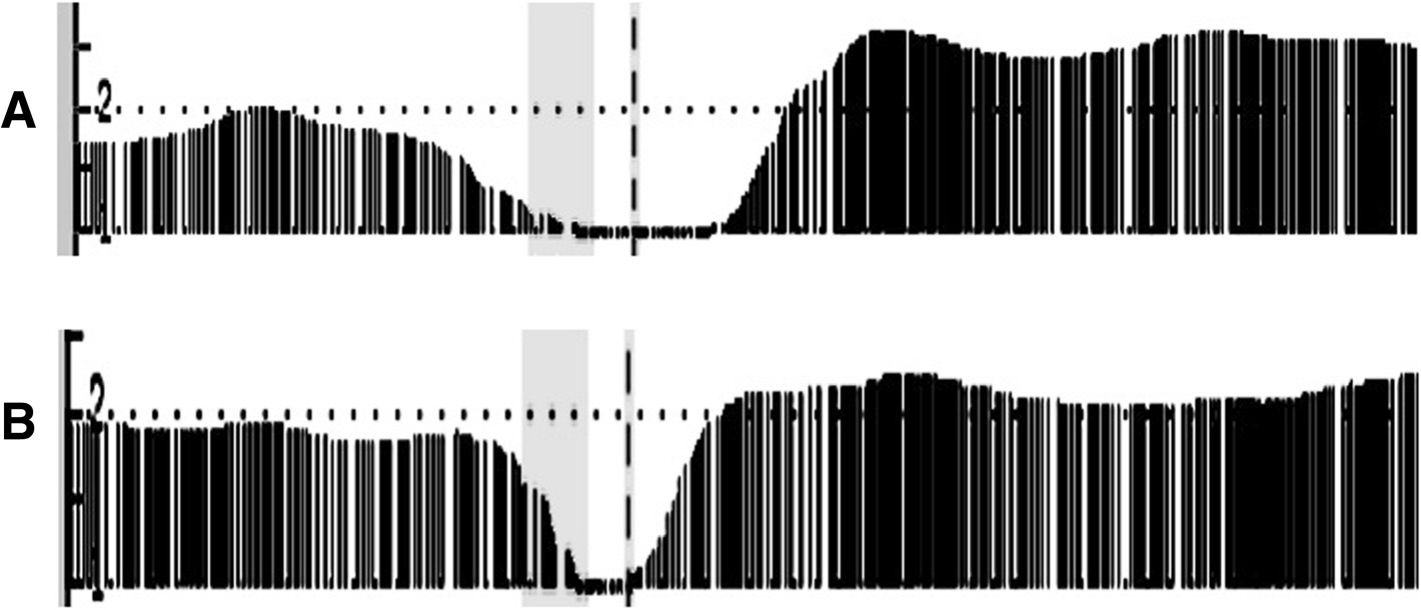
VHL locus (Ch.3 p25.3) deletion data display. The smooth signal derived from the raw data is displayed. The smooth signal value is calculated using the intensity values of the flanking probes and the data is displayed by a continuous line which is filled down to the x axis. Focusing on VHL locus was done by selecting the specific region of the chromosome and zooming in such that this region spans the entire x-axis. The data points containing the probe values are overlaid on the graph. Smooth signal value can be between zero or four depending on the Log(2) ratio raw values of the surrounding probes. **a** Sample 7 and (**b**) Sample ten smooth signal is one exhibiting deletion in *VHL* locus (labeled with dashed line). Probes around the area are normal (around 2)

We determined the *VHL* mutation status of our discovery cohort by both DNA sequencing and results from the CGH arrays. In two of the thirteen frozen samples (samples 7 and 10) *VHL* deletions were detected by our CGH analyses. The deletion in sample 7 encompassed 332 kb with 564 markers. The deletion in sample 10 was shorter (135 kb) with 344 markers. Figure 3 illustrates the smooth signal obtained in chromosome band 3p25.3 for these samples. Sequencing based mutational analysis of *VHL* did not reveal any additional mutations. Samples 7 and 10 incurred CNVs similarly to the other samples. The average number of CMVs in the other samples was seven. Sample 7 had 14 of the common CNVs thus had more than the average CNVs but sample 10 had less CNVs (six) which is similar to the average.

### CNV validation by digital droplet PCR

**Fig. 4:**
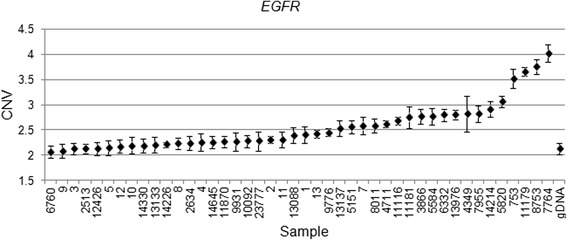
*EGFR* Copy number variation (CNV) values for clinical samples and one normal genomic DNA sample. Genomic DNA copy number alterations were assessed via ddPCR. The CNV is shown as the number of copies and the Poisson distribution at 95 % confidence interval. The copy number value of normal diploid sequence has a score of two. Copy number above two means amplification in that region and copy number below two means deletion in that region

**Fig. 5:**
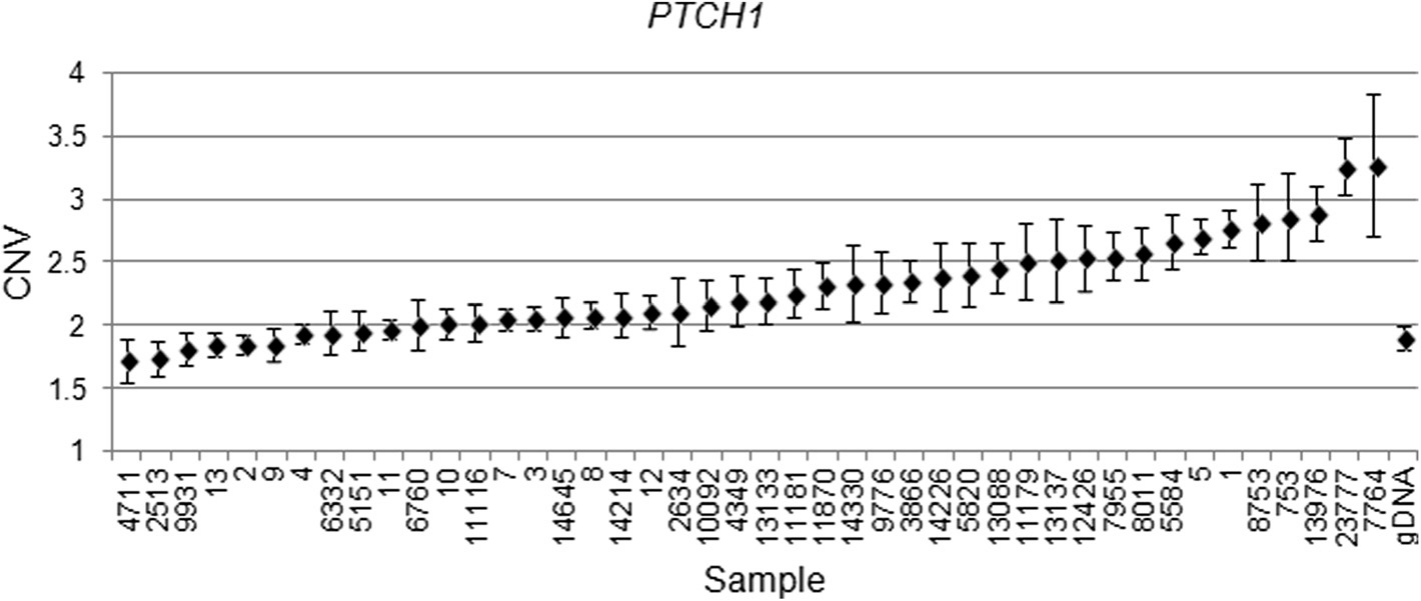
*PTCH1* Copy number variation (CNV) values for clinical samples and one normal genomic DNA sample. Genomic DNA copy number alterations were assessed via ddPCR. The CNV is shown as the number of copies and the Poisson distribution at 95 % confidence interval. The copy number value of normal diploid sequence has a score of two. Copy number above two means amplification in that region and copy number below two means deletion in that region

**Fig. 6:**
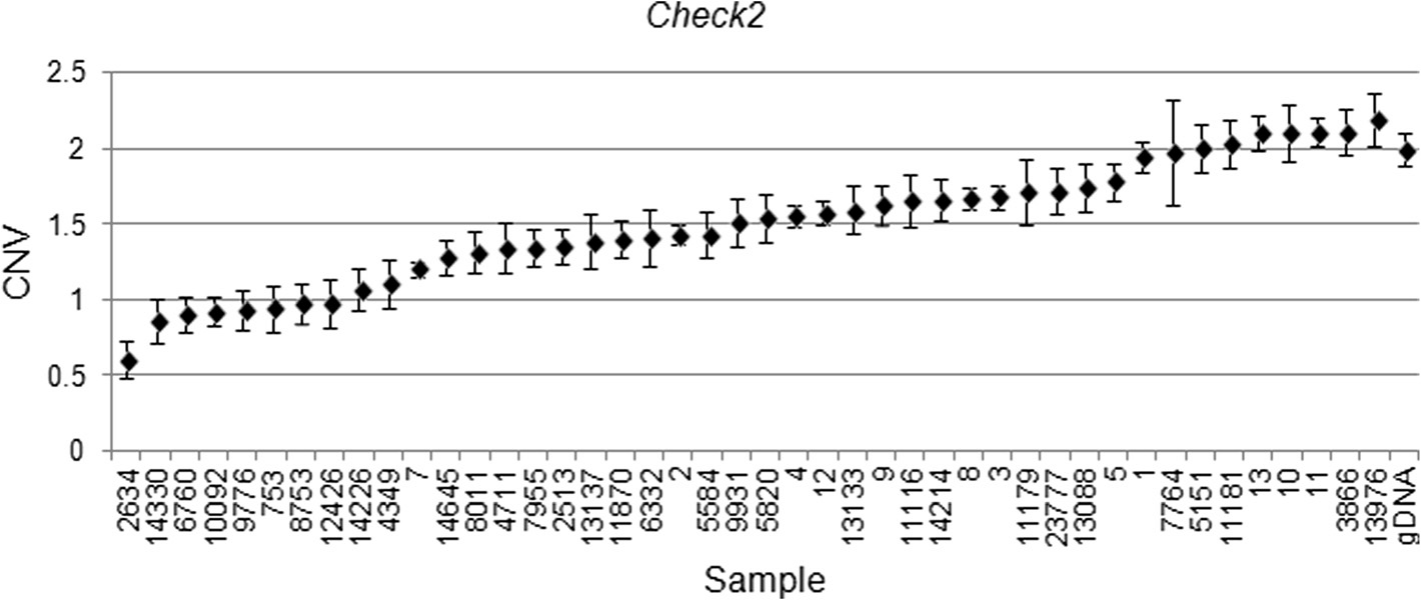
*CHEK2* Copy number variation (CNV) values for clinical samples and one normal genomic DNA sample. Genomic DNA copy number alterations were assessed via ddPCR. The CNV is shown as the number of copies and the Poisson distribution at 95 % confidence interval. The copy number value of normal diploid sequence has a score of two. Copy number above two means amplification in that region and copy number below two means deletion in that region

**Fig. 7:**
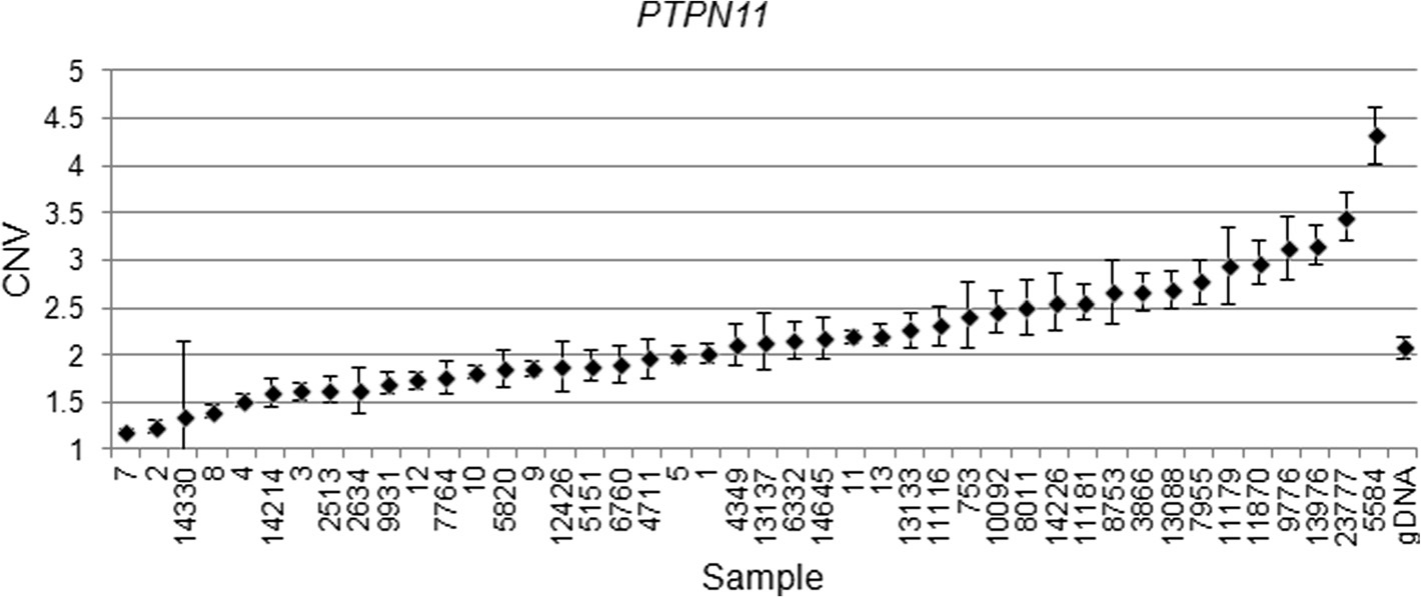
*PTPN11* Copy number variation (CNV) values for clinical samples and one normal genomic DNA sample. Genomic DNA copy number alterations were assessed via ddPCR. The CNV is shown as the number of copies and the Poisson distribution at 95 % confidence interval. The copy number value of normal diploid sequence has a score of two. Copy number above two means amplification in that region and copy number below two means deletion in that region

We validated our findings in a subset of four genes (*EGFR, CHECK2, PTCH1* and *PTPN11*) in the discovery cohort used for the array CGH and in an additional independent set of 32 FFPE specimens, using copy variation detection by digital droplet PCR (ddPCR) analysis. Samples were partitioned into thousands of nanoliter-sized droplets; single template molecules were amplified on a thermocycler, and counted for fluorescent signal. Absolute copy numbers of target and reference sequences were determined by Poisson algorithms [8]. The RNase P (Ribonuclease P) amplicon maps within the single exon *RPPH1* gene on 14q11.2 was used as the standard reference assay for copy number analysis [11]. Validation results were as follows: *EGFR* amplification was detected in 29 of 32 patient samples (Fig. 4), *PTCH1* was amplified in 18 of 32 patients (Fig. 5) and *CHEK2* was deleted in 27 of 32 (Fig. 6). Surprisingly, *PTPN11* was deleted in 7/32 whilst it was amplified in 17/32 specimens (Fig. 7).

It is important to note that in result of normal cell admixture within tumor samples a high CNV means either high fraction of tumor cells with relatively high copy number in each cell or low fraction of tumor cells with very high copy number in each cell. Likewise, low CNV means either high fraction of tumor cells with relatively low copy number in each cell or low fraction of tumor cells with relatively very low copy number in each cell. In short if the fraction of tumor cells is low then the relative copy number (both high and low CNV) is even higher than what is reported. In our cohort, histopathologic assessment by a pathologist determined that 47 percent of samples had more than 95 % tumor, 7 percent had more than 90 % tumor, 29 percent of samples had more than 70–80 % tumor, 14 percent of samples had between 50–70 % tumor and 3 percent had between 40–50 % tumor.

## Discussion

We report here for the first time a genome-wide, high-resolution systematic analysis of chromosomal changes in hemangioblastoma. Using the SNP array 6 (Affymetrix) we analyzed 1.8 million genetic markers genome wide to identify amplifications/deletions up to single gene level. We identified a total of 94 CNVs, 23 of them involved noncoding regions. 56 (31 gains and 25 losses) were found in at least two specimens. The most frequently gained regions were on chromosomes 1 (p36.32) and 7 (p11.2). The most frequently deleted region was on chromosome 12 (q24.13).

Our findings provide the first high-resolution genome-wide view of chromosomal changes in hemangioblastoma and identify 23 common, ie found in 4 or more patients, candidate genes for hemangioblastoma pathogenesis (Table 3): *EGFR, PRDM16, PTPN11, HOXD11, HOXD13, FLT3, PTCH, FGFR1, FOXP1, GPC3, HOXC13, HOXC11, MKL1, CHEK2, IRF4, GPHN, IKZF1, RB1, HOXA9, HOXA11* and several microRNA, including *hsa-mir-196a-2*. We note that some of these microalterations have been reported very rarely previously in tissue samples from healthy subjects (according to Database of Genome Variants (DGV), which are reported in Table 3. However, in our tumor samples these alterations are significantly more common (p < 0.00001).

Functional annotation analysis by David [12] reviled that two pathways are prominently affected in the hemangioblastoma samples: the cell proliferation and angiogenesis promoting pathways. The cell proliferation pathway includes the following genes: *CHEK2, EGFR, FGFR, FLT3* and *PTCH1*. The angiogenesis pathway includes the genes *EGFR* and *FGFR*, which are significant because they are also involved in blood vessel formation. Importantly, three of these genes were verified by ddPCR: *EGFR, CHEK2* and *PTCH1*. Furthermore, *PTPN11* was selected for verification as it was lost strikingly often (Table 3).

Epidermal growth factor receptor (*EGFR*) is a tyrosine kinase receptor that has been documented with increased expression in a variety of human cancers such as breast cancer, [13], early stage non-small-cell lung cancers and gliomas [14, 15]. Our findings are in line with three prior immunohistochemical studies that found *EGFR* overexpression in hemangioblastomas [16] [17] [18]. Fibroblast growth factor receptor 1 (*FGFR1*) micro deletions have been reported in myeloid and lymphoid neoplasms [19]. Interestingly we discovered that the *FGFR1* deletion involves the FGFR1 2nd intron (chromosome 8, 38291333–38314367), which according to UCSC genome browser hg19 includes a regulatory site. Checkpoint kinase 2 (*CHEK2*) has been implicated in DNA repair, cell cycle arrest, and apoptosis in response to DNA double-strand breaks [20]. Mutations in *CHEK2* have been reported to be possibly associated with breast cancer and liposarcoma development [21, 22]. The protein patched homolog 1 (*PTCH1*) is a sonic hedgehog receptor. Loss of function mutations in *PTCH1* are associated with development of various types of cancers, including medulloblastoma [23, 24], pancreatic cancer [25] and colorectal cancer [26]. In contrast to these observations, we find consistent *PTCH1* gains in our cohort of hemangioblastoma patients (Table 3). In support of our findings there are reports of *PTCH1* also acting as an oncogene in a mouse model of skin basal cell carcinomas [27].

FMS-like tyrosine kinase 3 (*FLT3*) plays a role in hematopoiesis including early hematologic differentiation and early B and T-cell development [28] and dendritic cells differentiation [29]. Activating *FLT3* mutations are some of the most common molecular abnormalities in acute myeloid leukemia (AML) [30]. Interestingly, in our data, we find losses of *FLT3* (Table 3). Consistent with our findings, others have reported deletions in *FLT3* in AML as well [31] [32]. Among the altered genes mapped in many of the recurrently gained regions we recognized several *HOX* genes (Table 3) and microRNAs (miRNAs) residing in the same region. *HOX* genes encode master transcription factors important in development and have been reported to be commonly altered in human solid tumors [33]. On the other hand, miRNAs are small regulatory RNAs that have recently been implicated in a variety of cancers [34]. miR-196a-2 and miR-196b gain were the most common miRNA CNV in our patients. Interestingly, miR-196a-2 differs from miR-196b by one nucleotide [35]. The miR-196 gene family is located in the regions of homeobox (*HOX*) transcription factors that are essential for embryogenesis. Up-regulation of miR-196a has been found in breast cancer, adenocarcinoma, leukemia and esophageal adenocarcinoma [35]. Accordingly the relevant causative change may actually be altered miRNA expression rather than *HOX* gene expression. Other studies have reported a 12-fold increase in miR-9 and a 15-fold decrease of miR-200a in hemangioblastomas distinguishing hemangioblastomas from metastatic clear cell renal cell carcinomas in the CNS [36]. *Prdm16* is preferentially expressed by stem cells throughout the nervous and haematopoietic systems and promotes stem cell maintenance [37]. Megakaryoblastic leukemia protein-1 (*MKL1*), is a transcription factor that regulates many processes, including remodeling of neuronal networks and epithelial-mesenchymal transition [38]. Moreover, deregulation by genetic alterations and/or altered *MKL1* transcription has been shown to have role in myeloproliferative neoplasms [39]. Finally and most interestingly, protein tyrosine phosphatase, non-receptor type 11 *(PTPN11*) gene encodes the tyrosine phosphatase SHP2 protein required for RTK signaling and has a role in survival, proliferation and differentiation [40]. The fact that we find the *PTPN11* gene is deleted in some hemangioblastoma patients and is amplified in others may suggest that these tumors actually originate from different cellular lineages. In fact, we found that spinal tumors were overwhelmingly deleted in the *PTPN11* region: 5 of 7 spinal tumors were deleted whilst 1 was amplified and 1 was chromosomally normal at this locus. In contrast, cerebellar hemangioblastomas were generally amplified: 16 out 37 were indeed amplified. However, 10 cerebellar tumors were deleted and 11 of 37 were normal in this genomic region. Interestingly, differential overexpression or deletion of *PTPN11* has been shown in other tumors. For example, mutations in *PTPN11* has been reported to be associated with development of Juvenile Myelomonocytic Leukemia (JMML) [41], acute myeloblastic leukemia (AML) [42], and acute lymphoblastic leukemia (ALL) [43]. Overexpression in gastric carcinomas has been reported [44]. In contrast, *PTPN11* has a tumor-suppressor function in liver [45] and cartilage [46]. Accordingly, decreased *PTPN11* expression was detected in a subfraction of human hepatocellular carcinoma specimens [45]. Thus, in contrast to its common pro-oncogenic role in hematopoietic and epithelial cells, *PTPN11* may act as a tumor suppressor in cartilage. Accordingly, we hypothesize that the *PTPN11* gene may act in a cell-specific manner: as a tumor suppressor on one hand in the progenitor cells of spinal hemangioblastomas, whilst it acts as an oncogene in the cells of origin of cerebellar hemangioblastoma tumors.

Previous analysis of six VHL-related CNS hemangioblastomas showed loss of chromosome 3p or the whole of chromosome 3 to be the most common abnormality, which is detected in 70 % and loss of 1p11-p31 in 10 % [47]. More relevant to our findings, published CGH studies on 10 sporadic cerebellar hemangioblastomas detected losses of chromosomes 3 (70 %), 6 (50 %), 9 (30 %), and 18q (30 %) and a gain of chromosome 19 (30 %) [48]. We indeed detected losses and gains in these areas but they were not frequent (15-20 %). Chromosome 3 losses were more abundant, mostly on p13 (5 samples *FOXP1*) and p25 (3 samples showed *PPARG* loss and 2 samples showed *VHL* loss). Interestingly, *FOXP1* transcription factor, located on chromosome 3(p13), can function as a tumor suppressor gene. Low expression in glioma [49] and Hodgkin lymphoma has been shown [50]. Differences in methodology, sample size and definition of aberration inclusion criteria may account for some of the apparent inconsistencies between previous studies and our findings. For example, previous results were obtained from VHL-related hemangioblastomas using techniques that identify deletions that are larger than 2 Mb. In the current study we used modern CGH microarrays which scan the DNA every 1 kb and thus we were able to identify very subtle genomic changes. One of the most striking observations in our study is that many CNVs affected single genes (Table 3) and revealed candidate genes, which have not been implicated in hemangioblastomas. This represents an important outcome of this study compared with previous investigations using CGH. Some of the new genes identified here as affected by CNVs in hemangioblastoma may serve as targets for future precisely targeted anti- cancer therapy. For example, antiangiogenic therapy can be given to patients with lesions that are not resectable.

## Conclusions

In this study, we have demonstrated in two different tumor cohorts and using two different techniques for copy number alteration detection, SNP and digital PCR, that *Chek2* is deleted and *EGFR*, *PTPN11, Ptch1* amplified in majority of hemangioblastoma patients. EGFR is the only gene that has been previously reported as a candidate gene with hemangioblastoma. Independent of HB tumor location *PTPN11* may act as tumor suppressor or oncogene depending on the tumor cell of origin. These findings have potentially relevant clinical value, as this the first high resolution for chromosomal alteration in HB. Future research should be dedicated to the prospective validation of these alterations and further characterization of tumors that carry the deletions/amplifications, as well as of defining the role of these genes. This may offer insights into hemangioblastoma biology, provide DNA-based markers that can be analyzed by FISH suitable for routine clinical applications and eventually lead to the development of effective targeted therapies for HB.

## Abbreviations

CNVs: Copy number variations
HB: Hemangioblastomas
CNS: Central nervous system
VHL: von Hippel-Lindau
CGH: Comparative genomic hybridization
SNP: Single nucleotide polymorphism
LOH: Loss of heterozygosity
ddPCR: Droplet Digital PCR
CNV: Copy number variation
FFPE: Formalin Fixed Paraffin Embedded
RNase P: Ribonuclease P
*EGFR*: Epidermal growth factor receptor
*FGFR*: Fibroblast growth factor receptor 1
*CHEK2*: Checkpoint kinase 2
*PTCH1*: Protein patched homolog 1
*FLT3*: FMS-like tyrosine kinase 3
AML: Acute myeloid leukemia
miRNA: microRNA
*MKL1*: Megakaryoblastic leukemia protein-1
PTPN11: protein tyrosine phosphatase non-receptor type 11
